# Optical Bioassays Based on the Signal Amplification of Redox Cycling

**DOI:** 10.3390/bios14060269

**Published:** 2024-05-24

**Authors:** Yunxiao Feng, Fengli Gao, Xinyao Yi, Ming La

**Affiliations:** 1 School of Chemistry and Environmental Engineering, Pingdingshan University, Pingdingshan 467000, China; 2743@pdsu.edu.cn; 2 College of Chemistry and Chemical Engineering, Anyang Normal University, Anyang 455000, China; 3 College of Chemistry and Chemical Engineering, Central South University, Changsha 410083, China

**Keywords:** redox cycling, colorimetry, fluorescence, surface-enhanced Raman scattering, chemiluminescence, electrochemiluminescence

## Abstract

Optical bioassays are challenged by the growing requirements of sensitivity and simplicity. Recent developments in the combination of redox cycling with different optical methods for signal amplification have proven to have tremendous potential for improving analytical performances. In this review, we summarized the advances in optical bioassays based on the signal amplification of redox cycling, including colorimetry, fluorescence, surface-enhanced Raman scattering, chemiluminescence, and electrochemiluminescence. Furthermore, this review highlighted the general principles to effectively couple redox cycling with optical bioassays, and particular attention was focused on current challenges and future opportunities.

## 1. Introduction

Sensitive and selective detection of chemical and biological targets is of importance in the fields of clinical diagnosis, environmental monitoring, and food quality control [1,2,3,4]. A variety of optical methods have been applied to determine various targets with high sensitivity and sensitivity, including colorimetry, fluorescence, surface-enhanced Raman scattering (SERS), chemiluminescence, and electrochemiluminescence [5,6]. Aiming to achieve excellent performances for the determination of low-abundance analytes, various signal amplification methods have been introduced into optical bioassays to improve the sensitivity, such as enzyme catalysis [7], nanomaterials [8,9,10], target recycling [11], nucleic acid amplification [12], and redox cycling [13]. Most of these methods are effective and sensitive but still suffer from some drawbacks for their practical applications. For example, natural enzymes have intrinsic limitations of high production cost and low tolerance to harsh conditions and operational stability, and target recycling and nucleic acid amplification methods require the use of tool enzymes.

Redox cycling is a process that can repeatedly produce or consume signaling species in the presence of reversible redox species or mediators and extra reductants or oxidants. In a typical system, redox cycling can be achieved electrochemically, chemically, or enzymatically based on repetitive oxidation–reduction reactions. Therefore, redox cycling can be simply coupled with other signal amplification methods, such as enzymes or nanocatalyst-driven chemical reactions and nanomaterial-based containers [14,15]. For example, electrochemical biosensors, by integrating redox cycling and enzyme catalysis, have been developed to show high sensitivity and signal-to-noise ratio [16,17,18]. Chemical–chemical redox cycling reactions have been successfully deployed in photoelectrochemical biosensors for the ultrasensitive detection of various biomarkers [19,20]. In electrochemistry-based heterogeneous biosensors, the electron transfer progress between the electrode and the substrate or mediator can be regulated by different factors, such as applied potential, conductive property of electrode, and scanning rate. Usually, the electron transfer rate between the electrode and the substrate or mediator is fast, and the chemical reaction rate between the reductant (or oxidant) and the substrate or electrode is relatively slow [21]. For the redox cycling-based homogeneous biosensors, it is difficult to exploit a matched couple of substrate, mediator, and reductant (or oxidant). To date, a few reviews have been reported to summarize the sensing mechanisms of electrochemical biosensors by coupling enzymatic catalysis and redox cycling [22]. Nevertheless, the signal amplification strategies in combination of different optical techniques and redox cycling reactions in homogeneous solutions have not yet been reported. To gain a comprehensive understanding of this field, we summarized the advances in optical bioassays based on the signal amplification of redox cycling, which are categorized by different signal output techniques, including colorimetry, fluorescence, SERS, and chemiluminescence. Particular attention was focused on the general principles, current challenges, and future opportunities.

## 2. Redox Cycling-Based Optical Bioassays

### 2.1. Colorimetric Methods

Colorimetric methods have become powerful tools for the detection of different targets due to their merits of cost-effectiveness, simplicity, real-time readout, and promise in point-of-care testing [23]. The conversion of chromogenic substances with the color change can be easily observed with the naked eye or quantified by a UV-Vis spectrophotometer. The introduction of a chemical redox cycling system can lead to the generation of abundant colored products, greatly improving the signal intensity. To achieve higher sensitivity, a series of strategies have been introduced into redox cycling-based colorimetric bioassays, including natural or artificial enzymes as labels and noble metal nanoparticles as novel chromogenic substrates.

**Figure 1:**
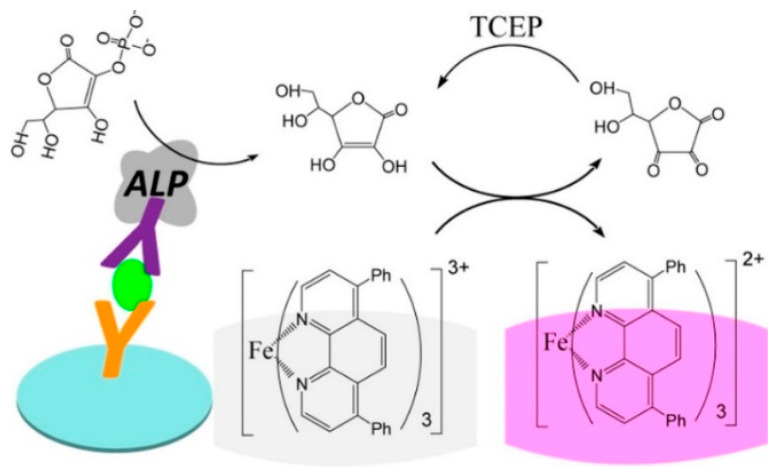
Schematic illustration of the redox cycling-based colorimetric ELISA for AFP detection [29]. Copyright 2019 American Chemical Society.

In the conventional colorimetric enzyme-linked immunosorbent assay (ELISA), enzymes such as horseradish peroxidase (HRP) and alkaline phosphatase (ALP) have been generally employed as the biocatalytic labels to trigger chromogenic reactions for signal output [24]. ALP can catalyze the dephosphorylation of ascorbic acid 2-phosphate (AAP) into ascorbic acid (AA), triggering the signal readout reactions for colorimetric assays [25,26]. The elegant convergence of redox cycling and classical enzyme-driven chromogenic reactions can significantly induce the color change and improve the sensitivity [27,28]. For example, Chen et al. reported the colorimetric ELISA of alpha-fetoprotein (AFP) based on the chemical redox cycling of AA [29]. As illustrated in Figure 1, ALP in the immune complex catalyzed the hydrolysis of AAP, and the colorless tris(bathophenanthroline) iron(III) (Fe(BPT)_3_^3+^) was reduced into pink red tris(bathophenanthroline) iron(II) (Fe(BPT)_3_^2+^) by the generated AA. In this process, AA was oxidized into dehydroascorbic acid (DHA) that could be rapidly reduced back into AA by tris(2-carboxyethyl)phosphine (TCEP). The repetitively redox cycling of AA resulted in the generation of abundant pink red Fe(BPT)_3_^2+^ complexes, significantly amplifying the colorimetric signal. In this method, the nonionic micelle of triton X-100 could encapsulate Fe(BPT)_3_^3+^ molecules, and the peripheral micelle and BPT prevented the reaction between the negatively charged TCEP and the positively charged Fe(BPT)_3_^3+^. The method showed a detection limit (LOD) down to 5 pg/mL, which was two orders of magnitude lower than that of the conventional ELISA.

**Figure 2:**
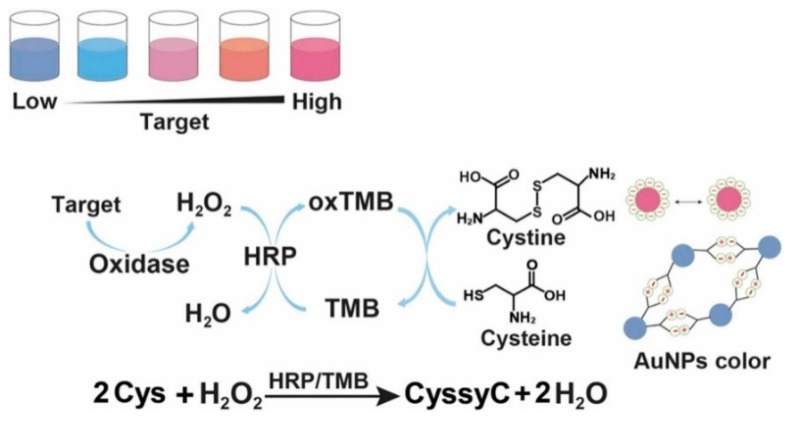
Schematic illustration of the plasmonic colorimetric bioassay based on the enzymatic cascade reaction and redox cycling of TMB [41]. Copyright 2023 Elsevier B.V.

Due to their outstanding characteristics such as high extinction coefficient and unique localized surface plasmon resonance (LSPR) property, gold nanoparticles (AuNPs) have been widely used as plasmonic substrates to construct various colorimetric sensing platforms [30,31]. Cysteine can stimulate the aggregation of AuNPs, and the LSPR coupling between the nearby AuNPs can induce the change of solution color from red to blue [32]. Oxidizing reagents such as H_2_O_2_ and O_2_ and enzyme catalysis can induce the oxidation of cysteine, thus indirectly modulating the aggregation of AuNPs [33,34]. However, the oxidant-aided direct oxidation of cysteine to cystine is a slow process. To improve the reaction rate between H_2_O_2_ or O_2_ and cysteine, several species have been be utilized as catalysts to accelerate the oxidation of cysteine, including I^−^ [35,36], Cu^2+^ [37], Fe^2+^ [38], and G-quadruplex/hemin chloride complexes [39,40]. In addition, substances with reversible redox activities can serve as redox mediators to develop redox cycling-based colorimetric methods. For instance, Liu et al. reported a label-free plasmonic biosensor for the colorimetric detection of H_2_O_2_ and cholesterol using HRP-assisted redox cycling of tetramethylbenzidine (TMB) (Figure 2) [41]. In the detection scheme, cholesterol oxidase catalyzed the oxidation of cholesterol to produce H_2_O_2_ that could oxidize TMB into blue oxidized TMB (oxTMB) in the presence of HRP. The generated oxTMB could be reduced back into TMB by cysteine. The repetitive regeneration of TMB continuously transmitted electrons between H_2_O_2_ and cysteine, causing the consumption of cysteine and limiting the aggregation of AuNPs. By integrating the redox cycling of TMB and cysteine-induced AuNP aggregation, this method showed a linear correlation in the concentration from 2 to 30 μM, with a LOD of 0.5 μM.

**Figure 3:**
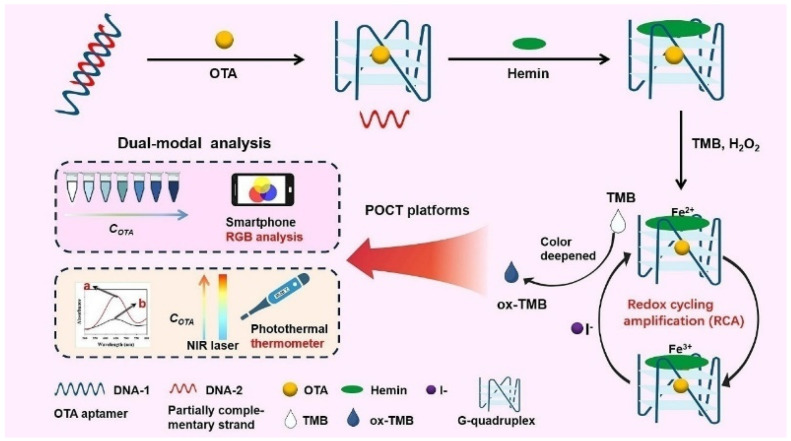
Schematic illustration of colorimetric and photothermal dual-mode aptasensor for the detection of OTA with redox cycling amplification [47]. Copyright 2023 Elsevier B.V.

Hemin, an iron-containing porphyrin compound, is a redox active cofactor of peroxidase [42]. It can be coordinated with cation-stabilized G-quadruplex to enhance the peroxidase-like activity [43,44,45,46]. As an HRP-mimicking DNAzyme, the hemin/G-quadruplex can catalyze the oxidation of chromogenic substrates by H_2_O_2_ via the Fenton reaction [43]. Tang et al. reported a colorimetric and photothermal dual-mode aptasensor for the detection of ochratoxin A with redox cycling amplification [47]. As shown in Figure 3, the presence of target OTA induced the formation of hemin/G-quadruplex DNAzyme that could catalyze the decomposition of H_2_O_2_ to form hydroxyl radicals for the oxidization of TMB. Meanwhile, Fe^2+^ ions in the porphyrin compounds were converted into Fe^3+^ ions. The resulting Fe^3+^ could be reduced back to Fe^2+^ by I^−^ that could repeatedly participate in the next Fenton reaction cycles. Thereby, the redox cycling of Fe^2+^-containing hemin/G-quadruplex induced the generation of numerous oxTMB species, resulting in the color change from colorless to deep blue. Under the irradiation of 808 nm near infrared (NIR) laser, the oxTMB molecules converted the signal into heat that could be monitored by a common thermometer. Under the optimized conditions, the developed dual-mode aptasensor exhibited low LODs of 1 pg/mL in colorimetric mode and 0.8 pg/mL in photothermal mode.

**Figure 4:**
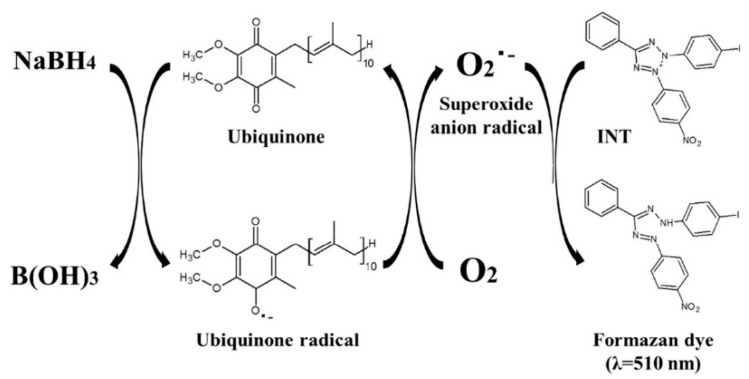
Schematic illustration of the reaction mechanism of colorimetric method for ubiquinone based on its redox cycle [51]. Copyright 2019 Elsevier B.V.

Quinones play an important role in maintaining biological functions of animal and plant, including ubiquinone (UQ) and pyrroloquinoline quinone (PQQ). Among them, UQ, a component of the mitochondrial electron transfer system, exists in the oxidized (ubiquinone) and reduced (ubiquinol) forms. It shows therapeutic efficiency for several diseases, including Parkinson’s disease [48], hypertension [49], and inflammation [50]. Thus, it is important to determine the concentration of UQ in biological samples. Fukuda et al. reported the ultrafast colorimetric microplate assay of UQ based on the redox cycling of quinine [51]. In the work, UQ molecules were reduced by NaBH_4_ to produce UQ radicals that could further reduce O_2_ into superoxide anion radical (O_2_^•−^). The generated O_2_^•−^ could oxidize the chromogenic substrate of 2-(4-iodophenyl)-3-(4-nitrophenyl)-5-phenyl-2H-tetrazoliumchloride (INT) into a pinked formazan dye with a strong absorbance at 510 nm [52]. The detailed reaction mechanism is shown in Figure 4. Under the optimized conditions, the method exhibited a linear range of 0.02–4 μM and a LOD of 14.8 nM.

**Figure 5:**
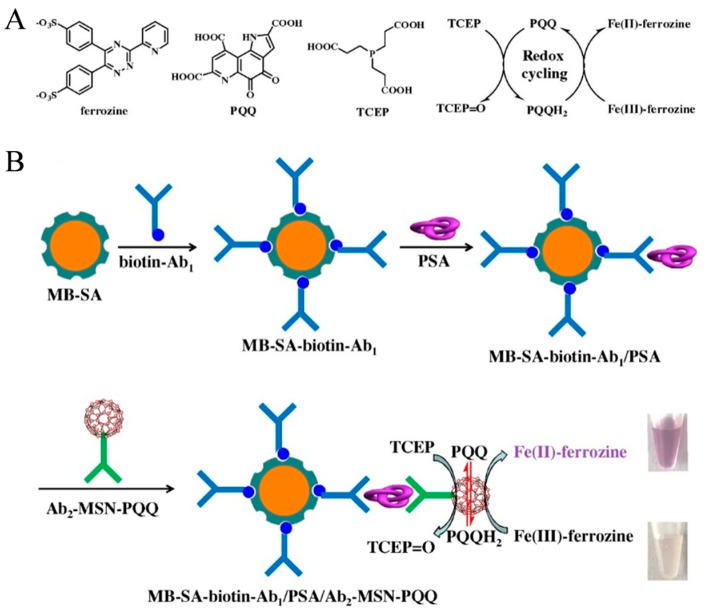
Schematic illustration of (**A**) chemical structures of ferrozine, PQQ, and TCEP and the proposed redox cycling; (**B**) MB-based colorimetric immunoassay of PSA by redox cycling with Ab2-MSN-PQQ as the nanolabel [57]. Copyright 2020 Elsevier B.V.

PQQ-modified nanomaterials have been exploited as redox mediators for the catalytic oxidation of TCEP and thiols [53,54,55,56]. For this consideration, Xia et al. reported the colorimetric immunoassay of prostate specific antigen (PSA) based on PQQ-initiated redox cycling between Fe^3+^-ferrozine and TCEP [57]. As displayed in Figure 5A, TCEP was used as the reductant to reduce PQQ into pyrroloquinoline quinol (PQQH_2_). The resulting PQQH_2_ could reduce the colorless Fe^3+^-ferrozine into dull red Fe^2+^-ferrozine. The redox cycling-based colorimetric system was coupled to the immunoassay for PSA detection. As shown in Figure 5B, streptavidin-modified magnetic bead (MB-SA) was used to load biotin-labeled capture antibody (biotin-Ab_1_). To improve detection sensitivity, mesoporous silica nanoparticle (MSN) was utilized as the nanocarrier for the loading of PQQ and recognition antibody (Ab_2_). After the sandwich-type immunoreaction and magnetic separation, PQQ contained in the nanolabel triggered the redox cycling in the presence of Fe^3+^-ferrozine and TCEP, leading to the generation of colorful Fe^2+^-ferrozine. With this method, PSA was detected in a linear range from 0.005 to 0.5 ng/mL with a LOD of 1 pg/mL.

**Figure 6:**
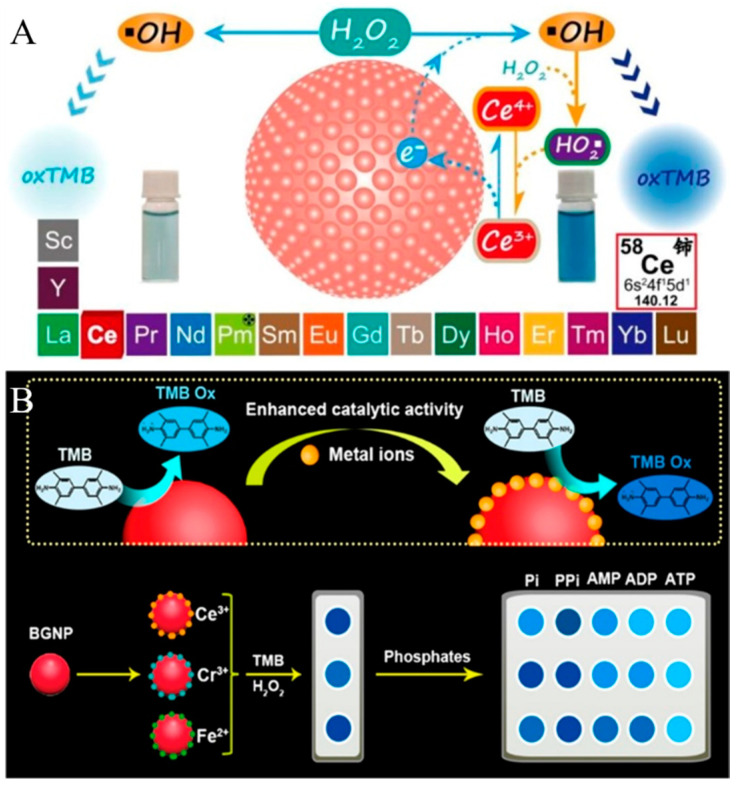
(**A**) Schematic illustration of the Ce^3+^-enhanced peroxidase-like activity of bare GNPs [64]. Copyright 2019 American Chemical Society. (**B**) Schematic illustration of the mechanism of screening sensing elements and fabrication of a colorimetric sensor array for the recognition of multiple phosphates based on the redox recycling-activated signal amplification of high peroxidase-like activity of bare AuNP–metal ion ensembles [65]. Copyright 2021 American Chemical Society.

It has been demonstrated that AuNPs show multiple enzyme-mimicking activities, including HRP, glucose oxidase, oxidase, catalase, and superoxide dismutase [58,59,60]. Although AuNPs have been widely used as nanozymes to develop versatile biosensors for the detection of various targets, the catalytic activity of AuNPs is relatively lower than that of natural enzymes or other nanozymes. Controlling surface chemistry is an effective approach to improve the catalytic activity of nanozymes because the nanozyme-catalyzed reactions mainly occur on the nanoparticle surface. For instance, metal ions (e.g., Hg^2+^, Pb^2+^, and Bi^3+^) can enhance the performance of AuNP-based artificial catalytic systems [61,62,63]. Deng et al. reported a colorimetric method for Ce^3+^ detection based on the redox recycling-triggered HRP-like activity enhancement of AuNPs [64]. As shown in Figure 6A, bare AuNPs exhibited relatively low catalytic activity and the addition of Ce^3+^ drastically increased the catalytic activity of bare AuNPs. The presence of AuCl^4−^/AuCl^2−^ on the surface of AuNPs facilitated the absorption of Ce^3+^ via electrostatic interaction. Then, Ce^3+^ donated the electrons to bare AuNPs for the generation of **^•^**OH. More importantly, the produced Ce^4+^ could react with HO_2_• to regenerate Ce^3+^ and stimulate the generation of **^•^**OH again. The redox cycling of Ce^3+^ greatly activated the catalytic activity of AuNPs. Based on this principle, this method was used to determine Ce^3+^ with a detection limit down to 2.2 nM. By taking advantage of metal ion-enhanced catalytic activity, Guan et al. used metal ion−AuNP ensembles to fabricate a colorimetric sensor array for the discrimination of multiple phosphates (Figure 6B) [65]. In the work, three metal ions (Ce^3+^, Fe^2+^, and Cr^3+^) were electrostatically adsorbed on the surface of bare AuNPs and greatly boosted the HRP-like activity. Because of the varied affinities between phosphates and metal ions, phosphates could be coordinated with metal ions to cause the different degrees of catalytic activity inhibition. This array could differentiate five phosphates at 0.25 μM based on the pattern recognition.

### 2.2. Fluorescence Assays

**Figure 7:**
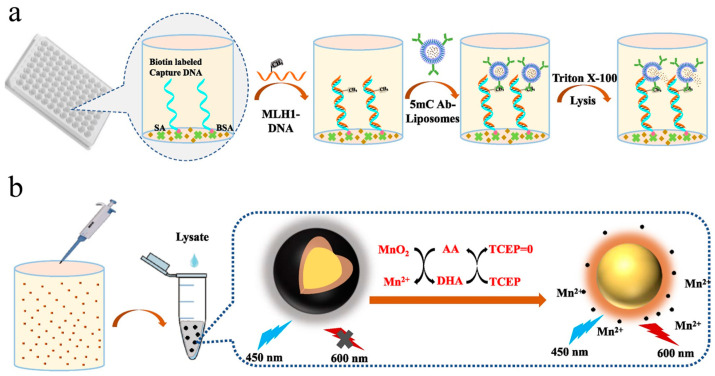
Schematic illustration of (**a**) procedure of methylated DNA detection and (**b**) liposome assisted chemical–chemical redox cycling signal amplification for methylated DNA detection [74]. Copyright 2023 Elsevier B.V.

Fluorescence assays have attracted much attention because of their high sensitivity, simple operation, and fast response. A variety of molecules and nanomaterials can be used as fluorescent probes or substrates, such as fluorescein isothiocyanate, semiconductor quantum dots, noble metal nanoclusters, and carbon dots [66]. AA can serve as a reducing agent to be electrochemically or chemically oxidized into DHA [67]. The regeneration of AA by TCEP or other reducing reagents has been coupled to different redox cycling strategies for electrochemical biosensors. Because of the high loading capacity, liposomes can encapsulate different signaling tracers and then serve as labels to improve the detection sensitivity, such as organic molecules, enzymes, and nanomaterials [68,69,70,71,72]. For instance, Zhang et al. reported the fluorescence immunoassay of DNA methylation, in which the generated AA by ALP enzymatic catalysis could trigger chemical–chemical redox cycling and improve sensitivity [73]. At the same time, they also constructed a universal fluorescent sensing platform for DNA methylation detection based on AA-carried liposome and redox cycling amplification (Figure 7) [74]. In this study, MnO_2_ nanosheets were coated in situ on the surface of Ru(bpy)_3_^2+^-loaded silica nanoparticles (Ru@SiO_2_@MnO_2_). The fluorescence of Ru@SiO_2_ nanoparticles was effectively quenched by MnO_2_ nanosheets via the inner filtration effect (IFE) due to the overlap of the respective absorption band and fluorescence emission band. Then, 5mC-antibody (5mC Ab)-conjugated and AA-encapsulated liposomes were used to recognize MLH1-mC DNA. The released AA molecules in the presence of TX-100 could reduce MnO_2_ nanosheets into Mn^2+^ ions, causing the fluorescence recovery of Ru@SiO_2_ nanoparticles. The resulting DHA (the oxidized form of AA) was reduced back to AA by TCEP through chemical–chemical redox cycling. The efficient regeneration of AA continued to etch MnO_2_ nanosheets, dramatically amplifying the fluorescence signal at 595 nm. With the aid of AA-encapsulated liposome and chemical–chemical redox cycling, the immunoassay exhibited a LOD down to 16.2 fM for the detection of methylated DNA.

**Figure 8:**
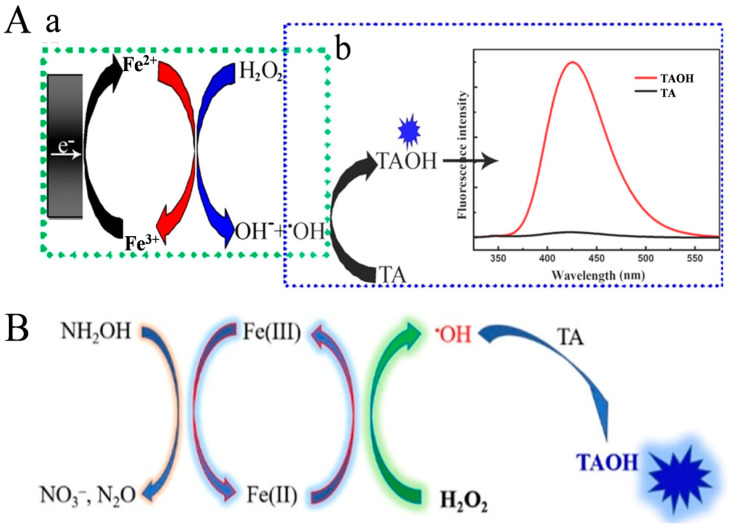
(**A**) Schematic illustration of (**a**) electrochemical–chemical cycling of Fe^3+^ generated •OH radical continuously and (**b**) •OH oxidized TA to highly fluorescent TAOH [77]. Copyright 2021 American Chemical Society. (**B**) Schematic illustration of the HA-enhanced Fenton reaction and •OH detection using TA [85]. Copyright 2022 American Chemical Society.

As a typical inorganic reaction, Fenton and Fenton-like reactions have been widely employed to improve the detection sensitivity because of their fast reaction rate, low cost, and mile reaction condition [75]. According to the reaction mechanism, the rate of Fenton reaction is severely restricted by the rate-limiting step over the reduction of Fe^3+^ to Fe^2+^. Therefore, the Fenton reaction can be accelerated by enhancing the Fe^3+^/Fe^2+^ redox cycling, eventually generating more hydroxyl radicals for signal output [76]. For instance, Zhang et al. developed a versatile and scalable method based on the electrochemical–chemical redox cycling of Fe^2+^ [77]. As illustrated in Figure 8A, Fe^3+^ was electrochemically reduced to Fe^2+^ at the electrode surface. The produced Fe^2+^ could activate H_2_O_2_ to generate ^•^OH. By electrochemical and chemical cycling of Fe^2+^, a large number of ^•^OH radicals were produced to oxidize terephthalic acid (TA) into highly fluorescent 2-hydroxyterephthalic acid (TAOH), enhancing the detection sensitivity. This method demonstrated a linear range from 5 fM to 100 nM, and the LOD for p53 detection was 1.7 fM. However, the requirement of both an electrochemical workstation and a fluorophotometer may limit the application of this method. It has been demonstrated that reductants can significantly promote the Fe^3+^/Fe^2+^ cycle in the Fenton reaction, boosting the production of ^•^OH radicals, including hydroxylamine [78,79,80], quinine [81], humic acid [82], and ascorbate [83,84]. For this view, Zhang et al. reported a simple and sensitive method for the detection of nucleic acid by fluorescence or naked-eye observation through chemical redox cycling amplification (Figure 8B) [85]. In the work, hydroxylamine hydrochloride (NH_2_OH·HCl, HA) was used to accelerate the Fe^3+^/Fe^2+^conversion, and TA was used as the ^•^OH trapping agent. Fe^3+^ at a small amount could be reduced into Fe^2+^ by protonated NH_2_OH (NH_3_OH^+^). The resulting Fe^2+^ could catalyze the decomposition of H_2_O_2_ via the Fenton reaction to generate plenty of ^•^OH radicals. In this process, Fe^3+^ was rapidly regenerated by protonated NH_2_OH to further activate H_2_O_2_. TA was oxidized by ^•^OH to produce fluorescent TAOH, which showed an emission peak at 425 nm under the excitation of 315 nm. Based on the Fenton-HA redox cycling system, the LODs were calculated to be 2.5 pM for HIV-DNA and 3 pM for miRNA-21.

**Figure 9:**
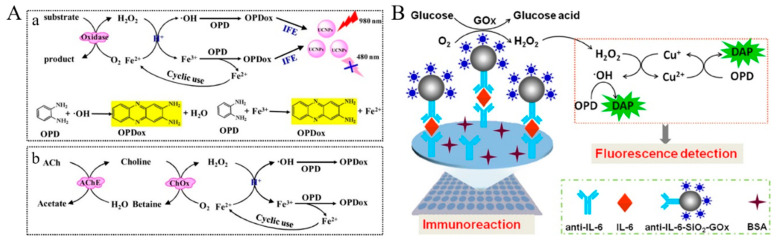
(**A**) Schematic illustration of fluorescence and colorimetric detection of H_2_O_2_-related analytes based on redox cycling signal amplification strategy (**a**) and an example for application demonstration (**b**) [86]. Copyright 2016 American Chemical Society. (**B**) Schematic illustration of the ultrasensitive split-type fluorescent immunoassay based on the enzymatic catalysis and Fenton-like reaction-triggered chemical redox cycling signal amplification [89]. Copyright 2023 Elsevier B.V.

Redox cycling of Fe^2+^ ions by fluorogenic substrates can increase the oxidation effect and improve detection sensitivity. Chen et al. constructed a fluorescent and colorimetric dual-readout method for the detection of H_2_O_2_-related analytes based on enzyme-controlled cyclic signal amplification [86]. As shown in Figure 9A, acetylcholinesterase (AChE) could catalyze the oxidation of acetylcholine (ACh) by O_2_ to form H_2_O_2_. The produced H_2_O_2_ could transform Fe^2+^ into Fe^3+^ and highly reactive hydroxyl radicals (•OH). Colorless *o*-phenylenediamine (OPD) could be concurrently oxidized into yellow color 2,3-diaminophennazine (denoted as OPDox or DAP) by the as-generated Fe^3+^ and •OH. Simultaneously, Fe^3+^ itself was reduced back to Fe^2+^ and then re-oxidized by H_2_O_2_ to produce more •OH radicals. Redox cycling of Fe^2+^ in the presence of H_2_O_2_ and OPD would produce a large number of OPDox, quenching the fluorescence of upconversion nanoparticles (UCNPs) based on the IFE effect. Meanwhile, the solution color changed from colorless to yellow, which was visualized by the naked eye for the direct quantification of choline and ACh. Furthermore, other metal ions, such as Cu^+^, Cu^2+^, and Co^2+^, can also serve as catalysts for the Fenton-like reaction to accelerate the decomposition of H_2_O_2_ [87,88]. For example, Chen et al. reported a split-type fluorescent immunosensor based on the enzymatic catalysis and Fenton-like reaction-triggered chemical redox cycling amplification (Figure 9B) [89]. In this study, Cu^2+^ serving as an oxidant could oxidize OPD into fluorescent DAP. The generated Cu^0^ in the format of nanoparticle in turn catalyzed the reaction between OPD and Cu^2+^ [90]. After the production of H_2_O_2_ by glucose oxidase (GOx) enzymatic catalysis, the formed Cu^+^ participated in the Fenton-like reaction to produce •OH and Cu^2+^, both of which could oxidize OPD substrates to generate more fluorescent DAP molecules. The regeneration of Cu^+^ via the OPD-mediated chemical redox cycling reaction initiated the Fenton-like reaction again, resulting in the remarkable increase in the fluorescence signal. This method could determine interleukin in a linear range of 20 fg/mL to 10 pg/mL with a LOD of 5 fg/mL.

### 2.3. SERS Methods

**Figure 10:**
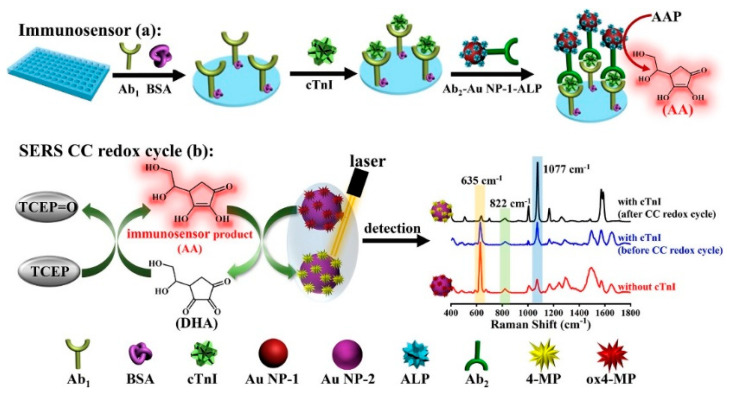
Schematic illustration of the CC redox cycle amplification-based ratiometric SERS immunoassay for cTnI detection [95]. Copyright 2023 American Chemical Society.

Surface-enhanced Raman spectroscopy (SERS) has aroused increasing research interest in analytical and biological chemistry because of its highly specific molecular fingerprint information, fast detection speed, and noninvasive and on-site analysis [91,92]. Nanostructured plasmonic materials, including gold, silver, and copper, can enhance the Raman signal via the electromagnetic enhancement mechanism. Ratiometric assay is a promising approach to improve the sensitivity and reproducibility in a complex sample matrix [93,94]. Zhao et al. developed a novel SERS platform for the sensitive detection of cardiac troponin I (cTnI) by integrating the redox cycling signal amplification with dual ratiometric immunoassay [95]. As illustrated in Figure 10, ALP in the cTnI-anchored sandwich structure catalyzed the generation of numerous AA molecules in the 96-well plate. After the transfer of the AA-containing solution to the SERS detection pool, the oxidized 4-mercaptophenol (ox4-MP) on the surface of AuNPs would be reduced into 4-mercaptophenol (4-MP) by AA. Simultaneously, the oxidation product (DHA) was reduced back to AA by TCEP, which can participate in the next reduction of ox4-MP. The redox cycling of AA by TCEP led to the production of more signal reporter 4-MP, causing the great change in the Raman signal between ox4-MP and 4-MP. The dual ratiometric-type SERS method achieved a linear range from 0.001 to 50 ng/mL and a LOD of 0.33 pg/mL (or 0.31 pg/mL) with the intensity ratio *I*_1077_*/*I_822_ (or *I*_633_*/*I_822_).

### 2.4. Chemiluminescence Assays

**Figure 11:**
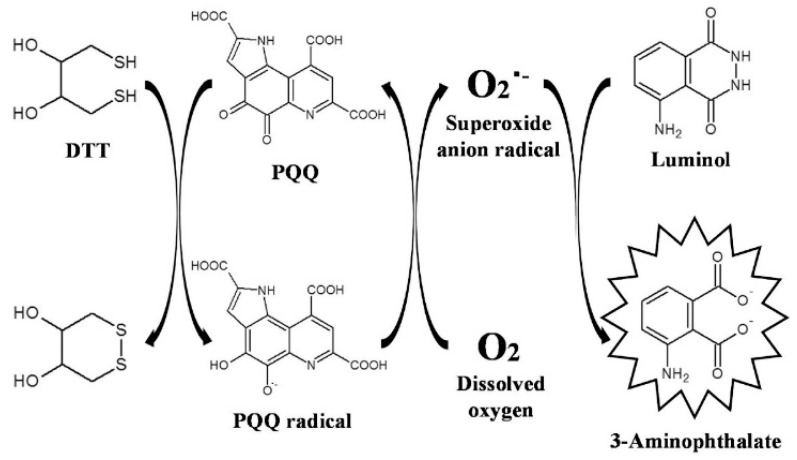
Schematic illustration of the mechanism of the chemiluminescence-producing reactivity of PQQ based on the PQQ redox cycle [103]. Copyright 2017 Elsevier B.V.

Chemiluminescence arises from the excited state of a species produced by a chemical reaction [96,97]. The method does not require the external excitation light source, thus avoiding the interference from light scattering and background emission from the sample matrix. Chemiluminescence has become one of the most popular detection tools in diverse fields because of its high signal-to-noise ratio and wide linear range [98]. Quinones can react with reductants such as thiol and NaBH_4_ to produce reactive oxygen species (ROS). The produced ROS can react with luminol to generate a chemiluminescence signal in the redox cycling [13]. Based on this principle, a variety of pharmaceutical quinines, including ubiquinone and doxorubicin, have been analyzed by chemiluminescence techniques [99,100,101,102]. For example, Fukuda et al. developed a chemiluminescence method coupled with liquid chromatography for the detection of PQQ, a cofactor for methanol dehydrogenase [103]. In the work, the solid phase extraction procedure was used to extract PQQ for chemiluminescence detection. The detailed mechanism is shown in Figure 11. Briefly, PQQ was reduced by dithiothreitol (DTT) to produce an unstable semiquinone radical that could react with dissolved oxygen to generate a superoxide anion radical (O_2_^•−^). The semiquinone radical was then oxidized into PQQ and the formed O_2_**^•^**^−^ reacted with luminol to produce a long-lasting intense chemiluminescence signal. PQQ was determined based on the redox cycling of PQQ in the presence of DTT. Based on the redox cycling of PQQ, the constructed chemiluminescence method for PQQ detection achieved a linear range of 4.0 to 400 nM with a LOD of 1.08 nM. In addition to the detection of quinones, Kishikawa et al. reported a chemiluminescence sensing platform for the determination and imaging of the tissue distribution of natural antioxidants [104]. In the work, quinines were reacted with antioxidants in natural sources to produce superoxide anions via redox cycling. The produced superoxide anions could react with luminal molecules to generate a long-lived and strong chemiluminescence signal.

**Figure 12:**
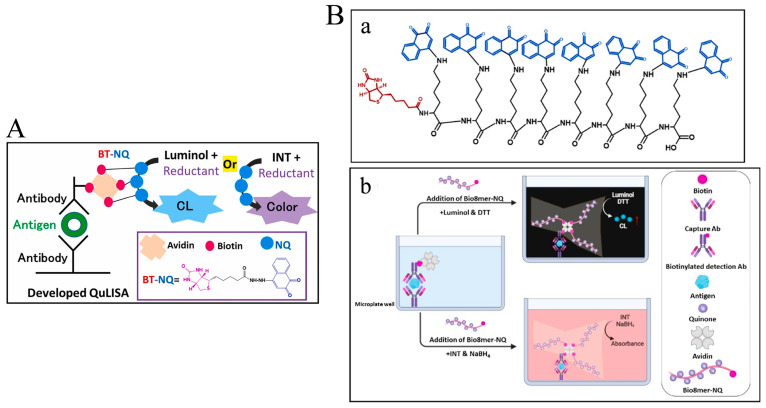
(**A**) Schematic illustration of the proposed QuLISA method with chemiluminescent and colorimetric detection based on the quinone redox cycle [107]. Copyright 2022 Elsevier B.V. (**B**) (**a**) Schematic illustration of the structure of Bio8mer-NQ and (**b**) the principle of multi-QuLISA [108]. Copyright 2023 Elsevier B.V.

Thanks to the interesting redox cycling, different quinone derivatives or quinone-loaded nanomaterials have been used and developed as the labels for the development of enzyme-free biosensors [105]. For example, El-Maghrabey et al. used 2-(9-carboxynonyl)-1,4-naphthoquinone (NQ) as the signal-generating tag to modify biotin (BT) and then developed a chemiluminescence method for avidin detection [106]. However, the synthesis of the signal label required a relatively long reaction time and a special catalyst. To address the problems, they reported a quinine-linked immunosorbent assay (QuLISA) for chemiluminescence assays using 1,2-naphthoquinone-4-sulfonate (NQS, Folin’s reagent) as a non-enzymatic tag (Figure 12A) [107]. NQS was reacted with BT-hydrazide without the use of additional catalysts. The redox cycling of NQS by DTT or NaBH_4_ could generate a superoxide anion radical to react with luminol or 2-(4-iodophenyl)-3-(4-nitrophenyl)-5-phenyl-2H-tetrazolium chloride (INT), producing an intense chemiluminescence signal or pink color, respectively. Nevertheless, the limited number of BT-NQ would decrease the detection sensitivity. For this view, Kaladari et al. used a branched oligomer dendrigraft poly-L-lysine generation 1 (DPLL G1) as a backbone to carry biotin and NQS (Bio8mer-NQ) for the fabrication of a multi-quinone-linked immunosorbent assay (multi-QuLISA) [108]. The structure of Bio8mer-NQ and the principle of multi-QuLISA are shown in Figure 12B. After the formation of sandwich-like immunocomplexes, quinones could generate a strong chemiluminescence signal or pink color through the redox cycle of quinone in the presence of DTT/luminol or NaBH_4_/INT, respectively. Under the optimized conditions, the food allergen, β-casein, was determined within a dynamic range of 78.1–2500 ng/mL with a detection limit of 3 ng/mL. Furthermore, dextran was utilized by Kuroda’s group to load biotin and water-soluble quinine anthracycline (doxorubicin) for signal multiplication [109].

**Figure 13:**
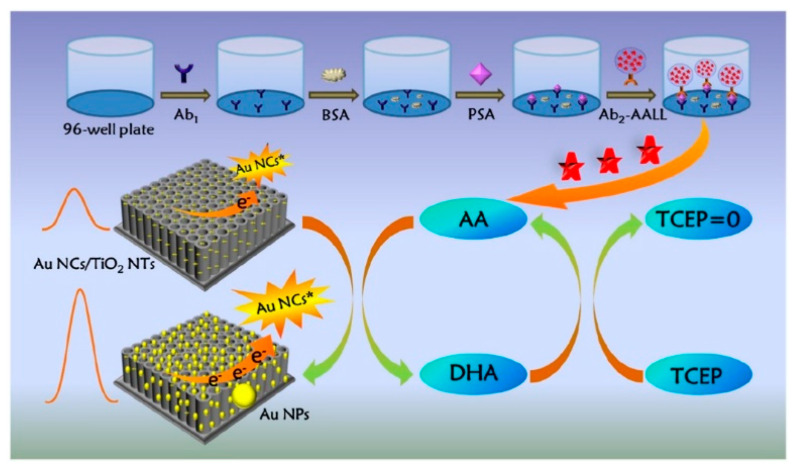
Schematic illustration of the split-type electrochemiluminescence immunoassay based on a liposome-assisted high-efficient chemical redox cycling strategy [117]. Copyright 2022 Elsevier B.V.

Electrochemiluminescence is a kind of luminescence that can emit from a molecular excited species formed at or near the electrode surface via electrochemical reactions [110]. On account of the fast analysis speed, good temporal and spatial controllability, and near-zero background noise, electrochemiluminescence bioassays have become a research hotspot in clinical diagnosis, food analysis, and environmental monitoring [111]. A large number of nanomaterials have been applied as the emitters for the development of electrochemiluminescence biosensors in recent years, including semiconductor quantum dots, silicon quantum dots, metal nanoclusters, and metal–organic frameworks [112,113]. Benefitting from the advantages of excellent light stability, ease of synthesis, and low toxicity, noble metal nanoclusters have been extensively used as electrochemiluminescence emitters, such as gold, silver, and copper nanoclusters, and so forth [114]. The electrochemical and chemical reduction of gold nanoclusters (AuNCs) can obviously enhance the electrochemiluminescence signal because of the close relationship between the valence state of Au and electrochemiluminescence intensity [115,116]. It is a feasible strategy to couple the redox cycling of reducing species with metal nanocluster-based electrochemiluminescence systems. For instance, Cao et al. developed a split-type electrochemiluminescence immunosensor for the detection of PSA based on liposome-assisted chemical redox cycling (Figure 13) [117]. The AA-encapsulated liposome was used to recognize the captured PSA. Then, a huge amount of AA was released under the treatment with TX-100. The lysates were transferred onto the electrode interface of AuNC-modified TiO_2_ nanotubes (TiO_2_ NTs). The chemical redox cycling of AA was initiated in the presence of Au^3+^ and TCEP. In this process, Au^3+^ ions were reduced in situ into AuNPs by AA on the electrode surface of AuNCs/TiO_2_ NTs, and the resulting DHA was then reduced back into AA by TCEP. The large loading capacity of liposomes and the advanced chemical redox cycling caused the continuous reduction of Au^3+^ ions, producing a large amount of AuNPs on the sensing electrode and significantly enhancing the electrochemiluminescence intensity. The method achieved a LOD down to 6.7 fg/mL and a linear range from 10 fg/mL to 10 ng/mL, Table 1.

## 3. Conclusions and Future Perspectives

Redox cycling reactions are relative to many common processes, e.g., the reactive centers or cofactors in natural enzymes, the generation of reactive oxidative processes, and multivalent metal ion-mediated catalysis. This review summarizes the recent efforts to integrate redox cycling reactions with versatile optical assays, including colorimetry, fluorescence, SERS, and chemiluminescence methods. Through the rational selection of extra reducing or oxidative agents, the redox cycling of mediators or targets can lead to the repeated generation of signal species for signal amplification. Thus, redox cycling endows the optical methods with high sensitivity, low LOD, and wide detection range. We introduced several representative examples for the determination of different targets and briefly discussed the detailed mechanisms. It is believed that redox cycling-based multiple signal amplification strategies are becoming an increasingly relevant alternative to traditional enzyme-based strategies, which will draw increasing attention from the sensor research community.

Although considerable progress has been made in the past few years, there are still some challenges that should be addressed in the future. First, the mechanisms regarding redox cycling-based assays are mainly based on the difference in reaction rates between extra reducing agents and signal species in presence or absence of mediators. Thus, interferences contained in the sample matrix or environment may affect the overall reaction. In this view, future research efforts should be made to decrease interference-induced false signals by exploiting appropriate reducing agents, mediators, and signaling species, and optimizing other conditions, such as temperature, pH, and activators or inhibitors toward enzymes. Second, most nanozyme-based catalysts involve redox cycling of multivalent metal ions on their surface. Although a growing number of nanozymes have been reported, their catalytic activity and substrate selectivity are still insufficient compared with natural enzymes. In this respect, the catalytic activity and efficiency of nanozymes should be enhanced by adjusting their size, shape, composition, and surface modification. The specificity of nanozymes can be increased by chemical recognition, such as molecular imprinted polymers and supramolecular chemistry. Third, optical assays employing nanomaterials as the signal indicators are increasingly reported due to the intrinsic properties of nanomaterials. Nanomaterials can be engineered to show accurate and controllable responsiveness to certain specific substances, and therefore the mediators or products in redox cycling can continuously and greatly affect their optical signal. Hence, integration of redox cycling and nanomaterials is still a striking direction in the development of optical assays.

## Figures and Tables

**Table 1:** Analytical performances of redox cycling-based optical methods.

Method	Redox Cycling System	Target	Linear Range	LOD	Ref.
Colorimetry	ALP enzymatic product AA in the presence of TCEP and Fe(BPT)_3_^3+^	AFP	0.01–5 ng/mL	5 pg/mL	[29]
	HRP-catalyzed oxidation of TMB in the presence of H_2_O_2_ and cysteine-mediated reduction of oxTMB	Cholesterol	2–30 μM	0.5 μM	[41]
	DNA/hemin complex in the presence of H_2_O_2_ and TMB	OTA	5 × 10^−3^–180 ng/mL	1 pg/mL	[47]
	Ubiquinone in the presence of dissolved O_2_ and NaBH_4_	Ubiquinone	0.02–4 μM	14.8 nM	[51]
	PQQ in the presence of dissolved O_2_ and DTT	PQQ	20–2.5 × 10^3^ nM	7.6 nM	[52]
	PQQ in the presence of extra reducing agent TCPE and Fe (III)-ferrozine	PSA	5 × 10^−3^–0.5 ng/mL	1 pg/mL	[57]
	Ce^3+^ in the presence of H_2_O_2_ and TMB on the surface of bare AuNPs	Ce^3+^	10–100 nM	2.2 nM	[64]
Fluorescence	ALP enzymatic product AA in the presence of TCEP and MnO_2_	DNA methylation	0.01–1 pM and 1–50 pM	2.9 fM	[73]
	ALP enzymatic product AA in the presence of TCEP and MnO_2_	DNA methylation	0.1–100 pM	16.2 fM	[74]
	Fe^2+^ in the presence of H_2_O_2_ on the electrode surface	p53	5 × 10^−6^–100 nM	1.7 fM	[77]
	Fe^2+^ in the presence of HA and H_2_O_2_	HIV-DNA and miRNA-21	0.01–10 pM and 0.01–30 nM	2.5 pM and 3 pM	[85]
	Fe^2+^ in the presence of enzymatic product H_2_O_2_ and OPD	ACh	0.2–1.5 μM and 1.5–160 μM	156.3 nM	[86]
	Cu^2+^ in the presence of enzymatic product H_2_O_2_ and OPD	Glucose	1 × 10^−3^–1 μM	0.3 nM	[87]
	Cu^2+^ in the presence of enzymatic product H_2_O_2_ and OPD	IL-6	0.02–10 pg/mL	5 fg/mL	[89]
SERS	ALP enzymatic product AA in the presence of TCEP and oxidized 4-mercaptophenol	cTnI	1 × 10^−3^–50 ng/mL	0.33 pg/mL and 0.31 pg/mL	[95]
Chemiluminescence	Ubiquinone in the presence of dissolved O_2_ and DTT	Ubiquinone	0.09–43.2 μg/mL	26 ng/mL	[99]
	Doxorubicin in the presence of dissolved O_2_ and N-(4-aminobutyl)-N-ethylisoluminol-lipoic acid	Doxorubicin	1–200 nM	0.17 nM	[100]
	PQQ in the presence of dissolved O_2_ and DTT	PQQ	0.3–6 μM	50 nM	[102]
	PQQ in the presence of dissolved O_2_ and DTT	PQQ	4–400 nM	1 nM	[103]
	Menadione in the presence of dissolved O_2_ and AA	AA	0.3–50 μM	0.18 μM	[104]
	Quinone in the presence of dissolved O_2_ and DTT	Biotin	1–100 μM	0.58 μM	[105]
	Naphthoquinonethe in the presence of dissolved O_2_ and DTT	Avidin	0.2–0.8 μM	23.4 nM	[106]
	Naphthoquinonethe presence of dissolved O_2_ and NaBH_4_	*β*-casein	78–2.5 × 10^3^ ng/mL	20.2 ng/mL	[107]
	Naphthoquinonethe presence of dissolved O_2_ and NaBH_4_	*β*-casein	78.1–2.5 × 10^3^ ng/mL	3.2 ng/mL	[108]
	Doxorubicinin the presence of dissolved O_2_ and NaBH_4_	biotinylated antibody	5–80 nM	0.55 nM	[109]
	Liposome-released AA in the presence of TCEP and AuNCs	PSA	1 × 10^−5^–10 ng/mL	6.7 fg/mL	[117]

Abbreviation: ALP—alkaline phosphatase; AA—ascorbic acid; BPT—bathophenanthroline; AFP—alpha-fetoprotein; HRP—horseradish peroxidase; TMB—3,3′,5,5′-tetramethylbenzidine; oxTMB—oxidized TMB; OTA—ochratoxin A; PQQ—pyrroloquinoline quinine disodium salt; DTT—dithiothreitol; TCEP—tris(2-carboxyethyl)phosphine; PSA—prostate-specific antigen; AuNPs—gold nanoparticles; OPD—*o*-phenylenediamine; HA—hydroxylamine; ACh—acetylcholine chloride; IL-6—interleukin-6; cTnI—cardiac troponin I; AuNCs—gold nanoclusters.

## Data Availability

Not applicable.
